# Expiratory Flow Limitation at Different Exercise Intensities in Coronary Artery Disease

**DOI:** 10.1155/2020/4629548

**Published:** 2020-05-21

**Authors:** Viviane Castello-Simões, Marlus Karsten, Vinicius Minatel, Rodrigo Polaquini Simões, Ester Silva, Nayara Yamada Tamburús, Ross Arena, Audrey Borghi-Silva, Aparecida Maria Catai

**Affiliations:** ^1^Department of Physical Therapy, Cardiovascular Physiotherapy Laboratory, Nucleus of Research in Physical Exercise, Federal University of São Carlos, São Carlos, São Paulo, Brazil; ^2^Department of Physical Therapy, Santa Catarina State University, Florianópolis, Santa Catarina, Brazil; ^3^Department of Physical Therapy and Integrative Physiology Laboratory, College of Applied Health Sciences, University of Illinois Chicago, Chicago, IL, USA

## Abstract

**Introduction:**

Expiratory flow limitation (EFL) during moderate intensity exercise is present in patients with myocardial infarction (MI), whereas in healthy subjects it occurs only at a high intensity. However, it is unclear whether this limitation already manifests in those with stable coronary artery disease (CAD) (without MI).

**Materials and Methods:**

Forty-one men aged 40–65 years were allocated into (1) recent MI (RMI) group (*n* = 8), (2) late MI (LMI) group (*n* = 12), (3) stable CAD group (*n* = 9), and (4) healthy control group (CG) (*n* = 12). All participants underwent two cardiopulmonary exercise tests at a constant workload (moderate and high intensity), and EFL was evaluated at the end of each exercise workload.

**Results:**

During moderate intensity exercise, the RMI and LMI groups presented with a significantly higher number of participants with EFL compared to the CG (*p* < 0.05), while no significant difference was observed among groups at high intensity exercise (*p* > 0.05). Moreover, EFL was only present in MI groups during moderate intensity exercise, whereas at high intensity all groups presented EFL. Regarding the degree of EFL, the RMI and LMI groups showed significantly higher values at moderate intensity exercise in relation to the CG. At high intensity exercise, significantly higher values for the degree of EFL were observed only in the LMI group.

**Conclusion:**

The ventilatory limitation at moderate intensity exercise may be linked to the pulmonary consequences of the MI, even subjects with preserved cardiac and pulmonary function at rest, and not to CAD per se.

## 1. Introduction

The literature shows us that cardiopulmonary exercise testing (CPET) has been increasingly used in many clinical settings to evaluate physical performance as well as the physiological mechanisms linked to exercise limitations, when present [1, 2]. In addition to the metabolic, cardiovascular, and traditional ventilatory parameters, we can identify, through a CPET, the presence of an expiratory flow limitation (EFL), which may lead to an increase in lung volumes that result in increased mechanical work and oxygen cost of breathing [3].

There are several ways to assess the EFL; among them, we quote the tidal exercise flow-volume loop, a method that evaluates the degree of limitation plotting the tidal exercise flow-volume loop according to a measured end expiratory lung volume obtained during exercise within the maximal flow-volume envelope obtained immediately prior to CPET at rest [2, 3]. During our literature research, we observed that this method of evaluation has been used by several investigators to better assess and quantify the degree of ventilatory constraints during exercise [4–12].

Analyzing a previous study of our laboratory [6] in which we evaluated EFL through the tidal exercise flow-volume loop in males who suffered a recent myocardial infarction (RMI) and compared with apparently healthy subjects, we observe that both during moderate and high intensity exercise, the subjects with myocardial infarction (MI) presented EFL, while the healthy subjects presented with an EFL only during high intensity exercise; however, that study did not evaluate individuals with stable coronary artery disease (CAD). In this way, we do not know if the EFL presented in MI patients during moderate intensity exercise is related to the CAD or is due to the consequences of MI. In this way, our objective was to assess the EFL during moderate and high intensity of aerobic exercise in patients with CAD (with and without MI), in order to test the hypothesis that MI is responsible for the development of an EFL at moderate intensity exercise, possibly due to changes in lung mechanics and ventilatory pattern that this population present, according to which the literature brings us [13, 14].

## 2. Materials and Methods

### 2.1. Design and Study Population

This is an observational, retrospective, cross-sectional, and comparative study. Men between 40–65 years of age were allocated in four groups: (1) RMI group, composed of participants with one episode of an uncomplicated MI (30–45 days of the event) treated with medications and/or coronary angioplasty; (2) late myocardial infarction (LMI) group, composed of participants with one episode of MI (6 months–3 years of the event) treated with medications and/or coronary angioplasty; (3) stable CAD group, composed of participants treated only with medication (without prior MI and/or coronary angioplasty); and (4) an apparently healthy control group (CG). All participants were invited to participate in this study that occurred between June 1, 2008, and April 25, 2013. The inclusion criteria for the first three groups were as follows: (1) preserved left ventricular ejection fraction (LVEF >50%); and (2) absence of unstable angina or significant cardiac arrhythmias and for the CG were as follows: (1) apparently healthy based on clinical examination. The inclusion criteria common for all groups were as follows: (1) nonsmoking; (2) normal respiratory muscle strength (maximal inspiratory pressure > 60% of predicted values) [15] and normal lung function [16]; and (3) sedentary lifestyle according to the Baecke questionnaire [17]. The exclusion criteria for all groups were as follows: (1) body mass index ≥30 kg/m^2^; (2) neurological, orthopedic, and musculoskeletal limitations that would preclude participation in the exercise protocols; (3) inability to perform the respiratory maneuvers during testing; and (4) functional capacity ≤4 metabolic equivalents.

This study was approved by the local research ethics board (Protocols 350/2007 and 180/2012), and all patients gave written informed consent.

### 2.2. Clinical Evaluation

Prior to study initiation, all participants submitted a previous clinical evaluation that consisted of: (a) anamnesis, (b) anthropometric measures, (c) resting 12-lead electrocardiogram (ECG) (Ecafix TC 500, São Paulo, São Paulo, Brazil), (d) maximal standard exercise test on a treadmill (DIGISTRESS Vega, Digitronica, Belo Horizonte, Minas Gerais, Brazil), and (e) laboratory measurements of glycemia, hemoglobin, lipid profile, urea, creatinine, and uric acid.

After clinical evaluation, the maximal walking velocity of each subject was identified on a treadmill (to be used in the CPET) (Master ATL, Inbramed, Porto Alegre, Rio Grande do Sul, Brazil), based on a previous study [18, 19].

### 2.3. Spirometric Measurements and Respiratory Muscle Strength

The spirometric measurements were assessed at rest to exclude individuals with obstructive and restrictive lung disorders. Additionally, these measurements were also obtained before each constant workload exercise test (CWET) [14] to acquire the maximal flow-volume loop that was used as reference for each exercise flow-volume loop; after each CWET, the pulmonary function test was repeated to assess the presence of bronchodilation due to exercise [3]. The forced vital capacity (FVC), forced expiratory volume in one second (FEV_1_), IC (inspiratory capacity), and maximal voluntary ventilation (MVV) were obtained using a spirometer (Med-Graphics CPX, St. Paul, Minnesota, United States) with a calibrated pneumotachograph according to ATS standardization [16]; the values obtained were compared to the predicted normal values of Knudson et al. [20]. Exclusion criteria to obstructive lung disease were set at a FEV_1_/FVC <0.70 (GOLD) [21] and to restrictive lung disease were set at a FEV_1_/FVC >0.70 and FVC <80 [22].

Furthermore, the respiratory muscle strength was assessed (MVD-300, GlobalMed, Porto Alegre, Rio Grande do Sul, Brazil) [1], and the values used to define maximal inspiratory pressure were those sustained for 1s and measured at residual volume [23]. The values obtained of maximal inspiratory pressure and maximal expiratory pressure were compared to the predicted normal values of Neder et al. [24].

### 2.4. Regular Physical Activity Pattern

Physical activity patterns were assessed through the modified Baecke questionnaire [17], in order to include, in the study, only subjects with sedentary lifestyle. This questionnaire consists of a scale of one to five (5 represents the most active) with eight questions pertaining to occupation, four addressing athletic activities and four addressing habitual leisure habits. Results are reported as a sum of scores (with a minimum score of 4.5 and maximum score of 14.5).

### 2.5. CPET

Symptom-limited CPET was performed on a treadmill (Master ATL, Inbramed, Porto Alegre, Rio Grande do Sul, Brazil). The ramping protocol was based on a previous study [18] (respecting the maximal walking velocity without running) with objective to identifying the value of ventilatory anaerobic threshold (VAT) [2, 25–28] of each participant. Throughout CPET, ECG (12 simultaneous leads), heart rate (HR) (WinCardio, Micromed, Brasilia, Distrito Federal, Brazil), and blood pressure (BP) were monitored and registered.

Ventilatory and metabolic variables were monitored and registered breath by breath in a metabolic cart (CPX-D/BreezeSuite 6.4.1, Medical Graphics, St Paul, Minnesota, United States), calibrated before each test according to the manufacturer's specifications. Data were analyzed after smoothed by moving averages of eight respiratory cycles [25]. Using the ventilatory method, three independent evaluators determined the VAT [25, 27, 28]. Oxygen uptake (VO_2_), VCO_2_ (carbon dioxide production), and respiratory exchange ratio (RER) (defined as the ratio between VCO_2_ and VO_2_) of the peak were expressed as the highest averaged values observed during the last 30 s of exercise [18, 19]. The minute ventilation (VE)/VCO_2_ slope was obtained by analyzing the linear relationship between VE and VCO_2_, with VE on the *y*-axis and VCO_2_ on the *x*-axis [29]. The breathing reserve was obtained and represents the ratio between VE and MVV, and both variables are in L/min.

### 2.6. Constant Workload Exercise Tests (CWETs)

Following a minimum resting period of 48 h after the CPET, two CWETs were performed (on a treadmill) to evaluate the EFL, at moderate and high intensity exercise. The loads applied during the tests (speed and inclination) were related to VO_2_ measured at VAT of the prior CPET. Moderate intensity corresponded to the values of VO_2_ in VAT minus 25%, and high intensity corresponded to the VAT plus 25% of VO_2_ [6]. Each test consisted of (1) 1 min in the standing position, (2) 4 mins of warm-up phase (at 3.0 km/h), (3) 10 mins at a constant workload, and (4) 1 min of active recovery at 3.0 km/h followed by 5 mins of passive recovery. During the last four minutes of each CWET, an IC maneuver was performed every minute (from functional residual capacity) to determine placement of tidal volume in order to obtain the exercise flow-volume loop. The tests were performed in ascending order of intensity, and the rest period between each test was 30 to 60 min [6]. Throughout CWETs, ECG (12 simultaneous leads), HR, and BP were monitored and registered.

Three exercise flow-volume loops representing each intensity (moderate and high) were selected by the visual method, and the best curve of the three was used for data analysis. The degree of EFL is defined as the percentage of tidal volume (obtained from the exercise flow-volume loop) that meets or exceeds the expiratory boundary of the maximal flow-volume loop [3], but in this study, a minimum of 5% of the tidal volume overlap was required for participants to be considered to have an EFL [5, 30]. Ventilatory and metabolic parameters were registered and analyzed as previously described in the subitem *2.5. CPET*. The variables obtained were as follows: VO_2_, VE, VE/VCO_2_, partial pressure of end-tidal carbon dioxide (P_ET_CO_2_), IC, and expiratory reserve volume (ERV); the inspiratory reserve volume (IRV) was calculated by the following equation: IRV = vital capacity − (ERV + tidal volume) [16].

### 2.7. Statistical Analysis

The sample size was calculated based on pilot study data using the degree of EFL as an endpoint (four volunteers in each group). To reach statistical significance (*p* < 0.05) at a power of 80%, a sample of 8 participants in each group was required (GPower software package, version 3.1.6, Kiel, Schleswig–Holstein, Germany). The Shapiro–Wilk test was used to investigate the data distribution. The chi-square test was used to compare the clinical data, risk factors, medications, and number of participants with EFL amongst groups, and Fisher's exact test was used to compare the clinical data pre- and posttreatment and number of participants with EFL between different loads of CWETs for the same group. One-way ANOVA (Tukey post hoc) was used to compare age, anthropometry, cardiac function, pulmonary function, respiratory muscle strength, and variables of CPET amongst groups. Two-way ANOVA (Tukey post hoc) was applied to compare the variables of CWETs and values of ERV and IRV amongst groups and between different loads of CWETs for the same group. The Kruskal–Wallis (Dunn post hoc) and Wilcoxon tests were used to compare the degree of EFL amongst groups and between different loads of CWETs for the same group, respectively. Stepwise regression analysis was performed to determine the possible influence of group, medications (beta-blockers, angiotensin-converting enzyme inhibitors, diuretics, hypoglycemic, lipid lowering, and antiplatelet/anticoagulant), and risk factors (past of smoking, hypertension, dyslipidemia, and diabetes) on the main studied variables (HR, VO_2_, number of participants with EFL, degree of EFL, ERV, and IRV). The level of significance was set at *p* < 0.05 for all statistical comparisons. All the analyses were carried out using the Statistica for Windows software release 5.1 (StatSoft, Inc, Tulsa, Oklahoma, United States).

## 3. Results

**Figure 1:**
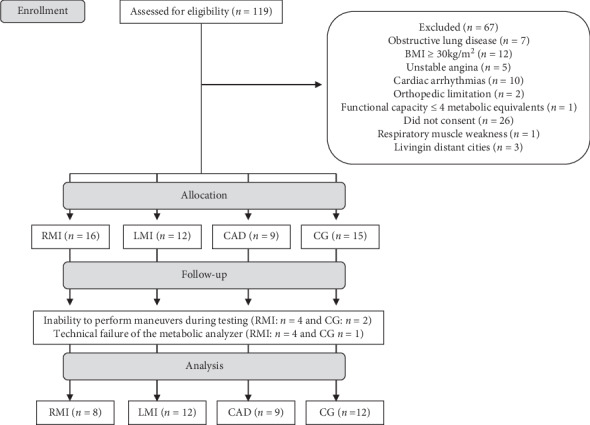
Flowchart showing subjects' participation in the study. n, number of individuals; BMI, body mass index; RMI, recent myocardial infarction group; LMI, late myocardial infarction group; CAD, stable coronary artery disease group; CG, control group.

Initially, 119 participants were recruited (95 were diagnosed with CAD, and 24 were apparently healthy subjects), 67 were excluded due to several factors (Figure 1), and, therefore, 52 were initially included in the study, being allocated into 4 groups. During evaluations, six presented inability to perform maneuvers during testing and five presented technical failure in the ventilatory and metabolic data and were excluded. Thus, 41 were included in the final sample (Figure 1).

Table 1 lists the age, anthropometry, risk factors, and medications. The majority of CAD participants had a risk factor profile; the CG used only lipid-lowering medication. In relation to the physical activity pattern, all participants were considered sedentary based on the Baecke questionnaire results, with a total score of or below 8 [17].

Table 2 shows the clinical data of the CAD and MI groups; there was no difference among groups regarding LVEF. In the CAD group, all participants were treated only with medication, according to inclusion criteria. The number of vessels affected and location of stenosis, both in pre and posttreatment conditions, are presented in Table 2. Some participants, even after undergoing the proposed treatment, remain with stenosis in less compromised arteries (3 participants in the RMI group and 7 in the LMI group).

Resting pulmonary function, respiratory muscle strength, and CPET variables are presented in Table 3. Regarding pulmonary function, the CAD and MI groups had lower values of MVV compared to the CG. During CPET, at VAT, the VO_2_ (mL·kg^−1^·min^−1^) was lower in the RMI group in relation to CAD group and CG; in the peak, the RMI group presented lower values of speed, VO_2_ (mL·kg^−1^·min^−1^), and HR in relation to CG; in relation to the LMI group, only speed was lower; the VO_2_ (mL·kg^−1^·min^−1^) at peak was too lower in the LMI group compared to CG.

Table 4 shows the CWETs variables at peak exercise (moderate and high intensity). At moderate intensity exercise, the speed was lower in the RMI group in relation to CG and CAD group; VO_2_ was lower in the RMI group compared to the CAD group and lower in the LMI group compared to CG and CAD group; lower P_ET_CO_2_ was observed in the RMI and LMI groups in relation to CG and CAD group. In relation to VE/VCO_2_, the RMI group presented with higher values compared to CG and the LMI group presented with higher values compared to CG and CAD group; HR was lower only in the RMI group in relation to CG. At high intensity exercise, the speed was lower in the RMI group in relation to CG and CAD group and lower in the LMI group compared to CG; only the RMI group presented with a lower HR in relation to CG and CAD group. When the different intensities were compared (high vs moderate), the following results were found: speed increased in the RMI group and CG, VE/VCO_2_ increased in the CG, P_ET_CO_2_ decreased in the CAD group and CG, and inclination of treadmill, VE, VO_2_, and HR increased in all groups.

**Figure 2:**
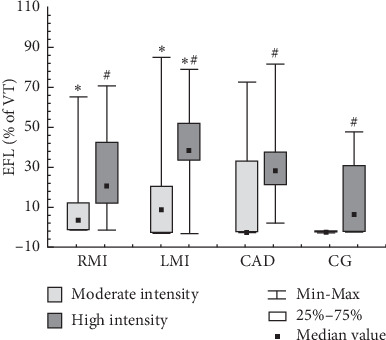
Degree of expiratory flow limitation at moderate and high intensity exercise. Data are presented as median ± (25%–75% and minimum–maximum). EFL, expiratory flow limitation; VT, tidal volume; RMI, recent myocardial infarction group; LMI, late myocardial infarction group; CAD, stable coronary artery disease group; CG, control group. ^*∗*^Difference in relation to CG in the same intensity. ^#^Difference in relation to moderate intensity exercise (*p* < 0.05). The Kruskal–Wallis (Dunn post hoc) and Wilcoxon tests were used.

During the moderate intensity of CWET, EFL occurred in 4/8 (50%) participants in the RMI group, in 7/12 (58%) participants in the LMI group, in 3/9 (33%) participants in the CAD group, and in none of the 12 participants in the CG. Only the RMI and LMI groups presented significant difference in relation to the CG. At high intensity of CWET, all studied groups presented with EFL and no significant difference was observed among them: 7/8 (87%) participants in the RMI group, 11/12 (92%) participants in the LMI group, 8/9 (89%) participants in the CAD group, and 7/12 (58%) participants in the CG. When the loads of CWETs were compared, the CAD group and CG presented a significant increase in the number of participants with EFL (high vs moderate).  Figure 2 shows the degree of EFL; at moderate intensity exercise, the RMI and LMI groups showed higher values when compared to the CG; during the high intensity, only the LMI group presented higher values when compared to the CG. When both loads of CWETs were compared (high vs moderate), all studied groups demonstrated a significant increase in the degree of EFL.

**Figure 3:**
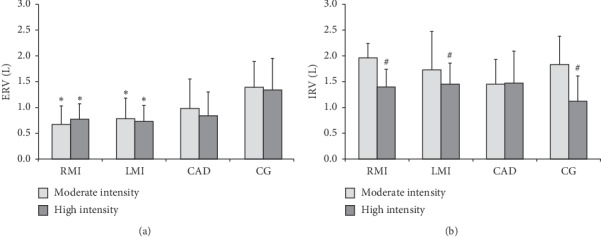
Expiratory reserve volume (a) and inspiratory reserve volume (b) at moderate and high intensity exercise. Data are presented as mean ± SD. EVR, expiratory volume reserve; IVR, inspiratory volume reserve; RMI, recent myocardial infarction group; LMI, late myocardial infarction group; CAD, stable coronary artery disease group; CG, control group. ^*∗*^Difference in relation to CG in the same intensity. ^#^Difference in relation to moderate intensity exercise (*p* < 0.05). Two-way ANOVA (Tukey post hoc) was used.

Figure 3(a) shows that the RMI and LMI groups presented with lower ERV values in both intensities in relation to the CG.  Figure 3(b) shows that the inspiratory reserve volume was lower in CG and MI groups at high intensity compared with moderate intensity.

A stepwise regression analysis was performed to determine the possible influence of group, medications (beta-blockers, angiotensin-converting enzyme inhibitors, diuretics, hypoglycemic, lipid-lowering, and antiplatelet/anticoagulant), and risk factors (past of smoking, hypertension, dyslipidemia, and diabetes) on the main studied variables (HR, VO_2_, number of participants with EFL, degree of EFL, ERV, and IRV). We observed that none of the variables were affected by medications and risk factors; however, the following influences were observed: (1) a number of participants with EFL and ERV at moderate intensity exercise were influenced by the group (*R*^2^ = 0.48, *β* = 0.48, and *p* < 0.001 and *R*^2^ = 0.48, *β* = −0.48, and *p* = 0.002, respectively) and (2) degree of EFL, ERV, VO_2_, and HR at high intensity exercise were influenced by the group (*R*^2^ = 0.41, *β* = 0,41, and *p* = 0.007; *R*^2^ = 0,46, *β* = −0,46, and *p* = 0.003; *R*^2^ = 0,44, *β* = −0,44, and *p* = 0.004; and *R*^2^ = 0,35, *β* = −0,35, and *p* = 0.027, respectively).

## 4. Discussion

The present study showed that subjects with stable CAD treated only with medication and no prior history of MI presented EFL only at high intensity exercise, which was similar to the apparently healthy participants, whereas participants with RMI and LMI presented with EFL at both moderate and high intensity exercises, suggesting that an EFL manifested at a lower workload may be a consequence of MI and not due CAD itself. Furthermore, another result obtained through of this study was that the EFL presented at moderate intensity exercise in patients with LMI (results similar to the RMI group), and highest degree of EFL at high intensity exercise compared to the CG suggested that a prolonged recovery time after MI was not been able to reduce the ventilatory limitation presented in this population.

In heart diseases, the presumable causes of EFL are related to changes in pulmonary mechanics, ventilatory pattern, and decreased respiratory muscle strength [13, 14]. Inspiratory muscle strength is a prognostic factor in heart failure and is an independent predictive factor for MI and death from cardiovascular diseases among the elderly [31–33]. In our study, however, no patient had respiratory muscle weakness at rest and there was no statistical difference among groups.

The EFL by the tidal exercise flow-volume loop was described in several studies with different populations [4–12], but to our knowledge, this is the first study that assessed the occurrence of EFL by the tidal exercise flow-volume loop in stable CAD participants without MI and/or coronary angioplasty during different exercise intensities (moderate and high) and compared responses those who have suffered a MI (RMI and LMI groups) and healthy participants. Our results have several clinical implications, as it may contribute to a better understanding of the ventilatory responses during this specific type of exercise in these populations (EFL patients with and without MI), leading to a more effective prescription of exercise training in order to reduce ventilatory limitations.

Additionally, our results showed that during moderate intensity of CWET, only RMI and LMI groups presented with lower values of ERV when compared to the CG and these results were also observed at high intensity exercise. Thus, we suggest that the presence of a ventilatory limitation found in MI participants is possibly related to the breathing strategy and a reduction of expiratory volumetric component during exercise. Even if this condition is compatible with dynamic hyperinflation and/or inefficient contraction of the expiratory muscles [3, 34, 35], due the methodologic limitations of the study, it was not possible to define the mechanisms responsible for the EFL observed.

Furthermore, the higher values of VE/VCO_2_ and lower values of P_ET_CO_2_ found in the MI groups, during moderate intensity of CWET, may be related to reduced ventilatory efficiency and the increased ventilatory demand [36] of these participants compared to controls and the CAD group. These factors may have contributed to the EFL, given that this limitation is related to the balance between demand and ventilatory capacity [3]. At the same time, other mechanisms, such as lung fluid accumulation, inflammation, alveolar-capillary membrane damage, narrowing of airway, and maldistribution of pulmonary ventilation and blood flow, may influence ventilatory function after acute MI [6, 37, 38].

Although the CAD and MI groups have shown lower values of MVV in relation to controls, in our study, this variable was not associated with the EFL found during the effort, since only the groups with MI showed an airflow limitation during the moderate intensity exercise. According to Johnson et al. [3], the use of MVV to estimate the available ventilatory capacity during exercise and to determine whether individuals have a mechanical limitation to their ventilation has many limitations. In the study by Klas and Dempsey [39], the authors showed that MVV does not represent the pattern typically observed during exercise, since the work of breathing is increased during exercise due to reflex hyperventilation.

Considering the results of the CG, we observed that the EFL occurs only at high intensity exercise, which was also showed in the previous study of Karsten et al. [6]. This response may be related to excessive ventilatory demands of exercise which leads to hyperinflation, a reduction in IC, and fatigue of the respiratory and skeletal muscles compromising oxygen transport. However, this response is expected, since healthy participants with superior exercise capacity requires greater ventilatory responses and the EFL is a consequence of this higher intensity during exercise [35, 40]. Furthermore, we observed that the same results were found in the CAD group, showing that these participants adopted a breathing strategy similar to the CG during both exercise intensities assessed.

Additionally, our results demonstrate that there is similarity amongst the CAD and MI groups regarding used medications (beta-blockers, angiotensin converting enzyme inhibitors, diuretics, lipid-lowering agents, and antiplatelet/anticoagulant agents), and risk factors (past of smoking, hypertension, dyslipidemia, and diabetes), while no influence on the main variables studied was observed, evaluated by stepwise regression analysis. Regarding a significant percentage of patients with history of smoking in the LMI group, we observed, during spirometric measurements, that these participants did not present changes in pulmonary function that could characterize an obstructive or restrictive lung disease; all of them presented FVC, FEV_1_, and FEV_1_/FVC normal values in relation to predicted values [20]. Regarding medications, it is important to emphasize that these are considered a standard therapy for these patients [41] given their use can contribute to improved prognosis [42]. Thus, the CAD and MI groups were under comparable medications, and only the CAD group did not present with an EFL at moderate intensity exercise. In this way, the influence of these factors were eliminated, again showing that the ventilatory limitation presented for MI groups (RMI and LMI) at a moderate intensity exercise may not be due the CAD but rather related to the ventilatory consequences of MI.

In our study, two CWETs were performed at moderate and high intensity, corresponding, respectively, on average to 50% and 80% of the VO_2_ peak (of CPET). As known, in a constant workload protocol at intensities between the 1^st^ and the 2^nd^ threshold (respectively, close to 40–60% and 60–80% of the VO_2_ peak) [43], the produced lactate can be buffered by bicarbonate, which would justify the noninterruption of exercise by the participants, thus allowing them to reach the predetermined time (10 minutes) [44].

Another important finding of this study in relation to the workloads applied during the two CWETs should be discussed; although the speed and inclination have been calculated according to VO_2_ corresponding to VAT of CPET, we observed that VO_2_ values obtained during the end of the two CWETs were above the expected values in all groups compared to the calculated VO_2_, that is, the moderate intensity corresponded to the VAT minus 25% of VO_2_, but the observed values were as follows: RMI group = minus 1%, LMI group = minus 10%, CAD group = minus 3%, and CG = minus 7%; the high intensity corresponded to the VAT plus 25% of VO_2_, but the observed values were as follows: RMI group = plus 49%, LMI group = plus 43%, CAD group = plus 29%, and CG = plus 54%. These results corroborate with those reported by Pithon et al. [45], in which healthy young subjects were evaluated during discontinuous step tests (70%, 100%, and 130% of VAT) and it is suggested that the workload should be 30% lower in the discontinuous protocol to reach an intensity close to VAT of incremental test. If we consider that during the incremental CPET, a temporal dissociation between what is being measured during expiration and what is happening in the muscle possibly could justify the higher VO_2_ values observed at moderate and high intensity in CWET relative to the expected VO_2_ (by calculation based on VAT) [46]. Additionally, we cannot fail to consider that during high intensity of CWET occurs, the slow component of VO_2_ kinetics appears, making this variable dependent not only on imposed workload but also on the time, thus explaining the higher VO_2_ values in this exercise intensity [47].

The main limitation of this study probably was the lack of the measurement of the indexes of end expiratory lung volume and end inspiratory lung volume, obtained through body plethysmography. These variables provide direct information about the ventilatory strategy and elastic load of the respiratory system during exercise [15]. Regarding cardiac function, the CAD and MI groups were characterized only by LVEF; as 69% was evaluated by ventriculography and only 31% by echocardiogram, it was not possible to assess the diastolic function of these patients. Another limitation is the different timing of MI of LMI group participants, since in this group there were participants with the time of the event ranging from 6 months to 3 years. Furthermore, this study did not include women because the selected age range could include both women with regular menstrual cycle (with and without use of contraceptives) and women in the postmenopausal stage (with and without use of hormone replacement therapy), as these differences could influence the results.

## 5. Conclusion

Participants with myocardial infarction (both RMI and LMI) presented EFL at moderate and high intensity exercise, different from participants with stable CAD that presented EFL only at high intensity exercise. Therefore, the presence of the ventilatory limitation at moderate intensity exercise may be related to the ventilatory consequences of MI and not due the CAD itself.

## Figures and Tables

**Table 1:** Age, anthropometry, risk factors, and medications of all groups.

	RMI (*n* = 8)	LMI (*n* = 12)	CAD (*n* = 9)	CG (*n* = 12)
Age, years	50.9 ± 5.5	54.7 ± 6.7	58.0 ± 4.4	51.8 ± 7.9
Anthropometry				
Height, m	1.68 ± 0.06	1.70 ± 0.08	1.69 ± 0.06	1.72 ± 0.05
Weight, kg	78.6 ± 10.2	83.4 ± 15.0	78.8 ± 10.0	76.5 ± 7.0
Body mass index, kg/m^2^	28.0 ± 4.3	28.7 ± 4.5	27.5 ± 2.3	26.0 ± 2.5
Risk factors, *n* (%)				
Past of smoking	1 (12.5)	8 (67)^*∗*^^+^	4 (44)	1 (8)
Hypertension	3 (37.5)^*∗*^	8 (67)^*∗*^	4 (44)^*∗*^	0
Dyslipidemia	7 (87.5)^*∗*^	10 (83)^*∗*^	8 (89)^*∗*^	3 (25)
Diabetes	3 (37.5)^*∗*^	5 (42)^*∗*^	2 (22)	0
Family history of CD	8 (100)	9 (75)	9 (100)	10 (83)
Medications, *n* (%)				
Beta-blockers	6 (75)^*∗*^	9 (75)^*∗*^	3 (33)	0
ACE inhibitor	3 (37.5)^*∗*^	8 (67)^*∗*^	2 (22)	0
Diuretic	3 (37.5)^*∗*^	5 (42)^*∗*^	0	0
Hypoglycemic	3 (37.5)^*∗*^	5 (42)^*∗*^	2 (22)	0
Lipid lowering	7 (87.5)^*∗*^	10 (83)^*∗*^	8 (89)^*∗*^	3 (25)
Antiplatelet/anticoagulant	8 (100)^*∗*^	12 (100)^*∗*^	6 (67)^*∗*^	0

Data are presented as mean ± SD or absolute value (percentage) of occurrence. RMI, recent myocardial infarction group; LMI, late myocardial infarction group; CAD, stable coronary artery disease group; CG, control group; *n*, number of individuals; CD, coronary disease; ACE, angiotensin-converting enzyme. ^*∗*^Significant difference in relation to CG. ^+^Significant difference in relation to RMI (*p* < 0.05). The chi-square test was used.

**Table 2:** Clinical data of groups with coronary artery disease.

	RMI (*n* = 8)		LMI (*n* = 12)		CAD (*n* = 9)
Time of MI, days	36 ± 14		512 ± 320		—		
Cardiac function					
LVEF, %	65.9 ± 6.1		68.8 ± 8.8		70.4 ± 10.0		
Type of treatment, *n* (%)					
Chemical reperfusion	1 (12.5)		1 (8.3)		—		
Mechanical reperfusion	6 (75.0)^*∗*^		10 (83.4)		—		
Only standard medication	1 (12.5)^*∗*^		1 (8.3)^*∗*^		9 (100)		
Number of vessels with stenosis, *n* (%)	Pre (*n* = 8)	Post (*n* = 8)	Pre (*n* = 12)	Post (*n* = 12)	
Without stenosis	1 (12.5)	5 (62.5)	0	5 (41.7)	0
One vessel	0	2 (25.0)	5 (41.7)	3 (25.0)	7 (77.8)
Two vessels or more	7 (87.5)	1 (12.5)	7 (58.3)	4 (33.3)	2 (22.2)
Location of stenosis, *n* (%)	Pre (*n* = 7)	Post (*n* = 3)	Pre (*n* = 12)	Post (*n* = 7)	
Anterior descending artery	7 (100)	2 (66.7)	7 (58.3)	0^*∗*^^#^	8 (88.9)
Left circumflex artery	4 (57.1)	2 (66.7)	8 (66.7)^*∗*^	6 (85.7)^*∗*^	1 (11.1)
Right coronary artery	4 (57.1)	0	5 (41.7)	3 (42.8)	2 (22.2)
Diagonal arteries	1 (14.3)	1 (33.3)	3 (25.0)	3 (42.8)	0
Marginal arteries	1 (14.3)	0	2 (16.7)	1 (12.5)	0

Data are presented as mean ± SD or absolute value (percentage) of occurrence. RMI, recent myocardial infarction group; LMI, late myocardial infarction group; CAD, stable coronary artery disease group; *n*, number of individuals; MI, myocardial infarction; LVEF, left ventricular ejection fraction; Pre, pretreatment; Post, posttreatment. ^*∗*^Significant difference in relation to CAD. ^+^Significant difference in relation to RMI. ^#^Significant difference in relation to pretreatment (*p* < 0.05). The chi-square test and Fisher's exact test were used.

**Table 3:** Resting pulmonary function, respiratory muscle strength, and CPET variables of all groups.

	RMI (*n* = 8)	LMI (*n* = 12)	CAD (*n* = 9)	CG (*n* = 12)
Pulmonary function				
FVC, % predicted	94.1 ± 9.6	98.8 ± 12.5	98.1 ± 10.1	101.9 ± 9.7
FEV_1_, % predicted	97.5 ± 13.4	96.8 ± 11.5	101.8 ± 6.4	100.9 ± 6.0
FEV_1_/FVC	0.83 ± 0.05	0.79 ± 0.06	0.80 ± 0.05	0.78 ± 0.03
MVV, L/min	145.5 ± 27.6^*∗*^	145.8 ± 25.1^*∗*^	142.4 ± 17.7^*∗*^	177.5 ± 30.4
Respiratory muscle strength				
MIP, cmH_2_O	96.0 ± 27.2	98.1 ± 14.1	97.1 ± 10.3	95.5 ± 21.6
MIP, % predicted	114.9 ± 5.4	111.6 ± 5.4	110.5 ± 5.8	113.8 ± 6.3
MEP, cmH_2_O	119.1 ± 31.7	131.4 ± 18.1	136.4 ± 19.6	139.7 ± 30.9
MEP, % predicted	121 ± 5.5	124.2 ± 4.4	120.4 ± 6.7	123.3 ± 6.4
**CPET**				
VAT				
VO_2_, mL·kg^−1^·min^−1^	13.5 ± 2.6^*∗*^^+^	15.6 ± 4.9	18.9 ± 4.2	18.6 ± 3.9
RER	0.87 ± 0.05	0.90 ± 0.04	0.87 ± 0.09	0.90 ± 0.04
Peak				
Speed, km/h	5.9 ± 0.4^*∗*^^+^	6.2 ± 0.5^*∗*^	6.4 ± 0.3	6.7 ± 0.3
Inclination, %	12.7 ± 4.7	14 ± 5.2	12.3 ± 4.8	17.2 ± 3.6
VO_2_, mL·min^−1^	1775 ± 383	1934 ± 409	1998 ± 461	2337 ± 543
VO_2_, mL·kg^−1^·min^−1^	23.2 ± 6.2^*∗*^	23.7 ± 5.6^*∗*^	25.4 ± 4.9	30.7 ± 6.0
VCO_2_, mL·min^−1^	2098 ± 423	2108 ± 504	2168 ± 412	2624 ± 597
VE/VVM	0.46 ± 0.08	0.44 ± 0.08	0.45 ± 0.17	0.45 ± 0.16
RER	1.18 ± 0.07	1.09 ± 0.09	1.10 ± 0.11	1.10 ± 0.05
HR, bpm	127 ± 20^*∗*^	143 ± 32	143 ± 17	165 ± 25
VE/VCO_2_ slope	32.4 ± 6.9	30.6 ± 3.9	26.6 ± 4.0	29.9 ± 4.3

Data are presented as mean ± SD. CPET, cardiopulmonary exercise testing; RMI, recent myocardial infarction group; LMI, late myocardial infarction group; CAD, stable coronary artery disease group; CG, control group; *n*, number of individuals; FVC, forced vital capacity; FEV_1_, forced expiratory volume in one second; MVV, maximal voluntary ventilation; MIP, maximal inspiratory pressure; ME, maximal expiratory pressure; VAT, ventilatory anaerobic threshold; VO_2_, oxygen uptake; RER, respiratory exchange ratio; VCO_2_, carbon dioxide production; VE, minute ventilation; HR, heart rate; VE/VCO_2_ slope, ventilatory efficiency. ^*∗*^Significant difference in relation to CG; ^+^Significant difference in relation to CAD (*p* < 0.05). One-way ANOVA (Tukey post hoc) was used.

**Table 4:** Constant workload exercise test variables (moderate and high intensity) of groups.

	RMI (*n* = 8)		LMI (*n* = 12)		CAD (*n* = 9)		CG (*n* = 12)	
	Moderate	High	Moderate	High	Moderate	High	Moderate	High
Speed, km/h	5.2 ± 0.8^*∗*^^+^	5.8 ± 0.4^*∗*^^+#^	6.0 ± 0.6	6.2 ± 0.5^*∗*^	6.2 ± 0.4	6.4 ± 0.3	6.4 ± 0.4	6.7 ± 0.3^#^
Inclination, %	0.1 ± 0.2	7.6 ± 2.7^#^	0.5 ± 0.9	10.1 ± 3.6^#^	0.9 ± 2.1	8.8 ± 5.2^#^	0.8 ± 1.7	11.1 ± 3.7^#^
VE, L.min^−1^	30.4 ± 5.4	54.1 ± 10.1^#^	38.3 ± 11.1	63.1 ± 19.9^#^	39.6 ± 9.5	65.8 ± 14.3^#^	36.6 ± 4.4	63.9 ± 12.2^#^
VO_2_, mL·kg^−1^·min^−1^	13.4 ± 2^+^	20.2 ± 3.3^#^	14 ± 3^*∗*^^+^	22.3 ± 5^#^	18.3 ± 4.5	24.4 ± 5.6^#^	17.3 ± 3.1	28.6 ± 4.6^#^
P_ET_CO_2_ (mL·kg^−1^·min^−1^)	38.4 ± 2.6^*∗*^^+^	37.5 ± 5.3	38.5 ± 1.4^*∗*^^+^	36.7 ± 4.6	44.1 ± 5.6	38.1 ± 4.7^#^	42.1 ± 2.2	38.7 ± 2.9^#^
VE/VCO_2_	30.1 ± 0.8^*∗*^	33.2 ± 4.6	31.0 ± 2.9^*∗*^^+^	34.2 ± 5.0	27.6 ± 1.9	29.7 ± 4.4	27.3 ± 1.8	30.2 ± 2.6^#^
HR, bpm	90 ± 9^*∗*^	112 ± 13^*∗*^^+#^	101 ± 16	127 ± 19^#^	115 ± 21	147 ± 18^#^	106 ± 14	139 ± 13^#^
IC, L	3.5 ± 0.5	3.6 ± 0.5	3.2 ± 0.5	3.3 ± 0.7	3.3 ± 0.2	3.3 ± 0.4	3.3 ± 0.7	3.3 ± 0.6

Data are presented as mean ± SD. RMI, recent myocardial infarction group; LMI, late myocardial infarction group; CAD, stable coronary artery disease group; CG, control group; VE, minute ventilation; VO_2_, oxygen uptake; P_ET_CO_2_, partial pressure of end-tidal carbon dioxide; VCO_2_, carbon dioxide production; HR, heart rate; IC, inspiratory capacity. ^*∗*^Significant difference in relation to CG in the same situation. ^+^Significant difference in relation to CAD in the same situation; ^#^Significant difference in relation to moderate intensity (*p* < 0.05). Two-way ANOVA (Tukey post hoc) was used.

## Data Availability

The data used to support the findings of this study are available from the corresponding author upon request.
